# Not so benign

**DOI:** 10.7554/eLife.42181

**Published:** 2018-10-26

**Authors:** Barbara Rivera

**Affiliations:** 1 Gerald Bronfman Department of OncologyMcGill UniversityMontrealCanada; 2 Lady Davis Institute for Medical ResearchJewish General HospitalMontrealCanada

**Keywords:** uterine leiomyoma, genome-wide association study, leiomyomagenesis, Human

## Abstract

Susceptibility to uterine fibroids, benign tumors that affect the health of many women, is linked to genes that are responsible for preserving genome integrity and promoting genitourinary development.

**Related research article** Välimäki N, Kuisma H, Pasanen A, Heikinheimo O, Sjöberg J, Bützow R, Sarvilinna N, Heinonen HR, Tolvanen J, Bramante S, Tanskanen T, Auvinen J, Uimari O, Alkodsi A, Lehtonen R, Kaasinen E, Palin K, Aaltonen LA. 2018. Genetic predisposition to uterine leiomyoma is determined by loci for genitourinary development and genome stability. *eLife* **7**:e37110. doi: 10.7554/eLife.37110

The established consensus is that benign tumors do not become malignant and do not metastasize, but this does not mean they are not clinically important. Uterine leiomyomas are a perfect example. These tumors, which are commonly referred to as fibroids or myomas, affect up to 70% of women before menopause (Stewart et al., 2017), and cause multiple clinical problems, including excessive uterine bleeding, pelvic pain, infertility and miscarriage. Fibroids are also the leading cause of hysterectomy, and therefore incur substantial medical costs around the world (Soliman et al., 2015).

Even in this cancer genomics era, our knowledge of the processes underlying the growth of these tumors is limited. Although they derive from a single smooth muscle cell in the uterine wall, fibroids are a heterogeneous tumor group. Broadly, they can be divided into three main groups, the most common being fibroids with alterations in *MED12* (a gene found on the X chromosome), followed by lesions that are deficient in an enzyme called fumarate hydratase, and lesions in which a protein called HMGA2 is overexpressed. Numerous risk factors contribute to fibroid development, including age, obesity, hormones, hypertension and ancestry, with African-American women at the top of the prevalence chart (Dvorská et al., 2017).

Multiple fibroids can occur as part of a hereditary syndrome called familial leiomyomatosis that is caused by germline mutations in the gene coding for fumarate hydratase. Moreover, and independent of familial leiomyomatosis, the first-degree relatives of affected women have a 2.5 times greater chance of developing fibroids than the general population. So, are there loci in the genome that are linked to a general susceptibility to leiomyomas? A handful of studies have explored this question and identified a few loci that are associated with a small increase in the risk of leiomyomas. However, these results are restricted to specific populations or have limited biological significance (Cha et al., 2011; Edwards et al., 2013; Hellwege et al., 2017). Now, in eLife, Lauri Aaltonen of the University of Helsinki and co-workers – including Niko Välimäki and Heli Kuisma as joint first authors – report how they have performed a genome-wide association study that sheds light on the development of leiomyomas (Välimäki et al., 2018).

The study was performed in five stages (Figure 1). In the first phase the researchers analyzed cases and controls from the UK Biobank, which resulted in a list of 50 candidate variants from 22 loci in the genome that might be associated with fibroids. These variants were single nucleotide polymorphisms (SNPs): that is, variants in which one nucleotide had been replaced by a different nucleotide. The researchers then computed the polygenic genomic risk score (GRS), which is a sum of the risk associated to each candidate variant, and validated the association of this score to fibroid development in an independent cohort from Helsinki. In the second phase the researchers combined the two populations in a case-control meta-analysis, which led to another seven candidate variants being identified (bringing the total to 57). The genomic risk score was recalculated in this second phase.

**Figure 1.**
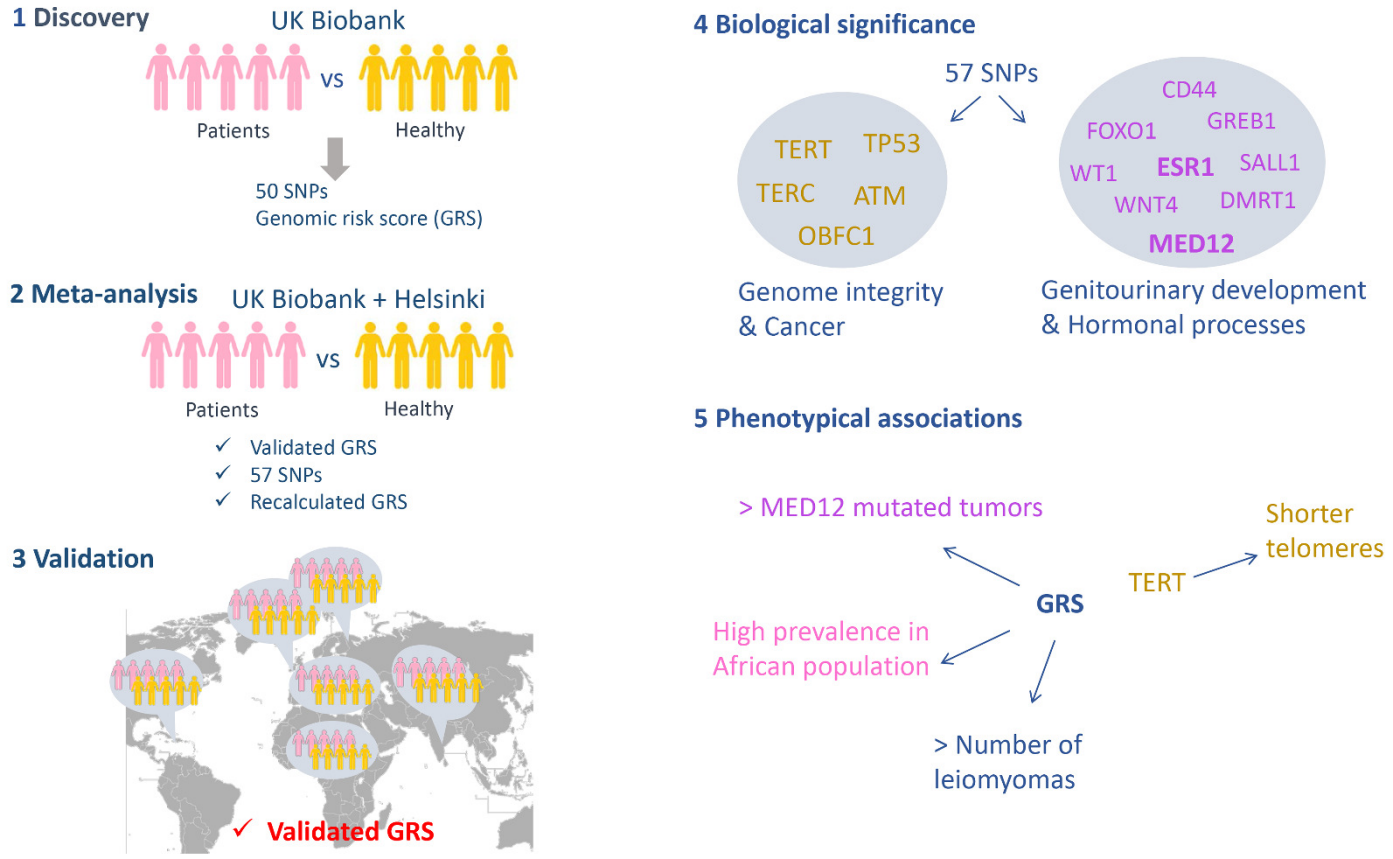
A genome-wide association study on leiomyomas. The study performed by Välimäki et al. had five phases. (1) Discovery: regions along the genome were compared in 15,453 women with leiomyomas (the cases) and a control sample of 392,628 women from the UK Biobank to identify locations where the two sets of genomes differed. 50 variants (in the form of a SNPs; single nucleotide polymorphisms) at 22 loci were identified and used to compute a genomic risk score (GRS). (2) Meta-analysis: Välimäki et al. then validated the risk score obtained in the first phase by studying cases and controls from Helsinki. Combining the UK Biobank and Helsinki samples lead to the identification of another seven variants. The risk was also recalculated at the end of this phase. (3) Validation: The risk score was then validated in six different ethnic groups (Northern Finland, Caribbean, African, Irish, Indian and 'other white'). When analyzed individually, only one SNP out of the total of 57 gave a significant signal in three different cohorts (UK Biobank, Helsinki and Northern Finland). (4) Biological significance: The genes located within the loci associated with leiomyomas can be divided into two groups: genes related to genome integrity and genes commonly altered in cancers (left), and genes related to the development of the genitourinary system and genes involved in hormonal processes (right). (5) Phenotypic associations: The risk score was associated with the number of leiomyomas per patient, with a higher prevalence of leiomyomas in African women, and with the presence of *MED12* mutations in the tumors. A SNP located within the *TERT* gene was associated with shorter telomeres.

In the third phase Välimäki et al. validated their candidates in six different ethnic groups. The genomic risk score was linked to the development of uterine leiomyomas in all six groups, confirming the involvement of these loci in the disease, independent of ethnicity. The researchers also identified an elevated risk in Black Africans compared to Irish (Caucasian), which could explain the differences in the leiomyoma prevalence.

In the fourth phase Välimäki et al. explored the biological significance of the loci they had identified by searching for genes located within these loci. The genes they identified could be classified into two groups: i) genes implicated in preserving the integrity of the genome and genes commonly implicated in cancer (such as *TP53*, which is sometimes called the 'guardian of the genome', and *TERT*, which helps to ensure that the ends of chromosomes remain intact during cell divisions); ii) genes related to the development of the genitourinary system and genes involved in hormonal processes, some of which are also deregulated in cancers (such as the gene for the estrogen receptor gene *ESR1*).

In the fifth phase the researchers showed that the risk score was positively associated with the number of leiomyomas per patient and a higher prevalence of the disease in African women compared to women with Caucasian ancestry. Overall the results are consistent with a recent study, by a different group, that compared cases and controls from the Icelandic population and Caucasians from the UK Biobank (Rafnar et al., 2018). Surprisingly, the results of Välimäki et al. showed that a variant called rs5937008, which is located in chromosome X near the *MED12* gene locus, predisposed women to the subtype of leiomyomas in which the *MED12* gene is altered. This result suggests that carriers of the rs5937008 variant have an increased risk of developing *MED12*-mutated leiomyomas in particular.

The study by Aaltonen, Välimäki, Kuisma and co-workers – who are based the University of Helsinki, Helsinki University Hospital, the University of Oulu and the Karolinska Institute – raises questions that are specific to leiomyomas. For example, what mechanisms orchestrate the process between susceptibility and tumorigenesis? There are also more general questions that remain unanswered: why, for example, do benign tumors – which can carry mutations similar to those seen in malignant tumors – remain benign (Crago et al., 2015; Agnihotri et al., 2016)? Answering such questions will benefit both research into leiomyomas and cancer research in general.
